# Intergenerational caring: a systematic literature review on young and young adult caregivers of older people

**DOI:** 10.1186/s12877-020-01976-z

**Published:** 2021-02-05

**Authors:** Barbara D’Amen, Marco Socci, Sara Santini

**Affiliations:** Centre for Socio-Economic Research on Aging, IRCCS INRCA - National Institute of Health and Science on Aging, Via Santa Margherita 5, 60124 Ancona, Italy

**Keywords:** Young caregivers, Young adult caregivers, Older people, Systematic literature review, Mixed methods appraisal tool (MMAT)

## Abstract

**Background:**

The theme of young family caregivers of older relatives is still partially uncovered, although the phenomenon is increasing worldwide. This Systematic Literature Review discusses methodological and content issues of ten articles covering this topic, in order to contribute to increase the knowledge and provide suggestions for designing effective support services for adolescent young caregivers. To this purpose, the findings of this review are framed within the caregiving stress appraisal model (renamed CSA model) elaborated by Yates’ and collegues, in order to highlight differences between young caregivers and the older ones.

**Methods:**

Multiple databases including PubMed, Web of Science, Scopus, ProQuest - Psychology Database, CINAHL Complete - EBSCOHost were used to carry out a systematic review of the literature. Additional references were retrieved from experts contacted and research knowledge. The selected articles underwent both methodological appraisal and contents analysis: for every article an appraisal score was calculated and themes and sub-themes were identified.

**Results:**

Out of the ten included studies three were mixed methods, six qualitative and one quantitative. Nine reached a high quality methodological score and one medium. Four main themes emerged from the content analysis: aspects of the caregiving relationship; effects of caregiving; coping strategies; recommendations for services, policy and research.

**Conclusions:**

Selected studies explored practical features of the relationship between young caregivers and older family members (tasks performed, motivations, coping strategies) and highlighted both positive and negative outcomes on young people’s everyday life condition and future development. Nevertheless, these evidences were often limited to small samples that did not allow to make generalizations. More studies are needed including large samples in order to deepen the different aspects of caregiving and design tailored support services.

## Background

Family caregivers, or informal or unpaid caregivers, provide 80% of long-term care in Europe, representing the bulk of health and social care to older or disabled people [1]. About 17% of the population in Europe [2] and 18.2% in the U.S. [3] is responsible for providing long-term care to older and disabled relatives.

Recent demographic and epidemiological changes, e.g. the extension of life expectancy and the increasing share of older people with multiple chronic diseases, might determine the growth of health and social care demand, thus increasing the number of family caregivers needed [2]. In the family context, the provision of care can be considered as a continuum starting with caring about i.e. with low levels of care responsibility, moving on to taking care, i.e. increasing care responsibility, up to providing intense and regular assistance. According to the literature, family caregivers are classified as “primary caregivers”, i.e. persons who provide the majority of caregiving tasks [4–7]; “secondary caregivers”, i.e. carers who assist primary caregiver in making decisions and complete his/her hands-on care [8]; “tertiary caregivers”, i.e. carers providing periodical and additional support to primary caregiver and do not make decisions on the cared recipient but help with issues do not directly concerning healthcare e.g. grocery and homework [9]. Finally, “auxiliary caregivers” provide an extra-support to primary caregivers for bettering the assistance: they provide companionship to the care recipients (e.g. grandparents), try to meet their emotional needs and participate in social activities with them [10].

As regards the tasks, the family caregivers perform, on a voluntary basis, a wide range of activities requiring different levels of effort, from company to help in carrying out the activities of daily living (ADLs) up to psychological and emotional support. The effects of care on different life realms of caregivers have been largely documented by the literature, such as anxiety and depression [11], relational strains [12], social isolation that may lead to perceived stress and loneliness [13]. Moreover, providing care and exposure to the suffering of a loved one can increase the risk for psychological and physical morbidity [14].

Despite the increasing number of male caregivers [15] the primary family caregiver is typically an adult and almost always a middle-aged woman [16, 17]. Nevertheless, in developed countries several changes in the labour market and in family settings, e.g. increasing number of employed women, lack of strong family networks, living in single parent families [18] can turn young people into family caregivers. Sometimes, young family caregivers help adult relatives, i.e. their parents, and provide assistance to a frail or disabled family member, e.g. grandparent or sibling [19] thereby playing the role of auxiliary caregivers [20, 21]. However, parents might need care themselves because of mental illness and/or physical disability and, in this case, young children have to take on the role of primary caregiver [19, 22, 23].

The definition of young caregiver differs across countries, according to the level of awareness of the civil society and of research carried out on the topic [24]. In the literature, there are different interpretations concerning the age brackets identifying a caregiver as young. In fact, there are more [25–27] and less extensive interpretations of the age range identifying both young caregivers [28, 29] and young adult caregivers [19, 25]. This heterogeneity could make it difficult to compare findings from different studies, as deepened in the Methods section.

A second difficulty is the low level of self-awareness; many youngsters, indeed, do not recognize themselves as family caregivers [30, 31]. This can happen because their culture of affiliation takes it for granted that they have to cover this role in the family, or because caring is considered as an extension of family relations [32]. Poor self-awareness may lead to a third problem: identification, which could entail resultant difficulties for recruitment and enrollment of young caregivers in research and support programs [18].

Even though there is still a dearth of quantitative cross-national studies on young family caregivers, several statistics and surveys at national level provided important information for grasping the dimension of the phenomenon, though taking into account different age ranges and, moreover, not allowing a full comparability of findings. For example, in the U.S. one fifth of caregivers was aged between 18 and 34 [3], in Canada over 1 million youth between the ages of 15–24 years (28.2% of the whole population in that age range) provided some kind of unpaid child and elder care [33], while in Australia one in twenty people (5.6% or 151,600 persons) aged 15–24 years were young caregivers [34].

Concerning Europe, in the last decade a growing attention has been paid to young caregivers, particularly in the UK, where, in 2011, there were 178,000 unpaid young caregivers (5 to 17 years-old), i.e. 19% more than in 2001 [35]. A recent cross-national survey [36] carried out in six European countries (Italy, the Netherlands, Slovenia, Sweden, Switzerland, and the UK) showed that out of 9,298 respondents, 28% were adolescent young caregivers (aged 15–17). A further exploration of the same database showed that 16.9% of adolescent caregivers aged 15–17 were caregivers of grandparents [37], suggesting the need for proofing the experience of young and adolescent caregivers of older relatives. Regardless of the age of the care recipient, when in the household a situation of disability and/or a chronic health condition occurs, young people may increase their level of involvement in carrying out basic domestic chores such as cleaning and tidying and they can start to help family members in need of care perform the activities of daily living e.g. dressing, eating, washing, up to provide support through medical care [38, 39]. When the person in need of care is old, young people are pushed to provide care by the unavailability or unwillingness of the adult family members. Thus, young people provide care for contributing to the family ecosystem and/or in response to a request of parents, especially when the latter are working caregivers [40].

Young caregivers of older care recipients perform a wide range of caring activities: personal hygiene and meal preparation [40, 41], help for instrumental activities of daily living, companionship and emotional support [42].

If, as highlighted above, the care activity can have a negative impact on the physical and mental health of adult family caregivers, this can happen all the more to young people and adolescents who, being still in a developmental age, can present psychological and emotional fragility [41]. According to the literature, young caregivers identify significant worries and problems in relation to their well-being, and these come over and above any ‘normal’ adolescent difficulties [43]. In particular, they report bad physical health [44], high levels of stress [45], fear and nervousness [46]. Moreover, they can run the risk of depression [47] and mental illness [48] and experience health inequalities, social, educational and employment exclusion [28, 49, 50]. These findings are enriched by studies that compared young adult caregivers with their non-caregivers peers, in which caregivers had significantly higher levels of symptoms of depression and anxiety than non-caregivers [51]. Following this comparative approach between young adult caregivers and non-caregiving peers, one study stated that young adult caregivers appear to be at risk for impairment in sleep quality, which in turn might impact health [52]. Furthermore, young caregivers reported less reliance on problem-solving coping, higher somatization and lower life satisfaction if compared with non-caregivers [53]. Nevertheless, several studies pointed out even possible positive effects of caregiving on adolescent and young people, e.g. learning coping skills for the future, feelings of gratification, a closer relationship with the cared for person [42] and a greater empathy [29]. A recent study comparing the impact of caregiving among adolescent young caregivers of grandparents to adolescent caregivers of other care recipients (i.e. other relatives and friends) [37] showed that the quality of the relationship between the young caregiver and his/her grandparent can mitigate the negative impact of caregiving, e.g. frustration, sense of inadequacy and mental health problems. Despite the increasing number of young people providing assistance to their relatives across the world, the aforementioned studies representing an exception, young family caregivers are not sufficiently considered by the literature, especially those caring for older family members with functional disability, in most cases grandparents [21, 37].

## Objectives and conceptual framework

The main goal of this systematic literature review is to cover this gap in knowledge by exploring how scientific literature treats the topic of young and young adult caregivers of older relatives, from a methodological as well as a content-based perspective. Hence, a methodological appraisal was carried out and the findings of selected articles were then analyzed, in order to reply to the main research questions, as suggested by Petticrew et al. [54]:

1. What are the methodological characteristics of the articles included in this review?
2. What are the main findings that emerge from these studies?
3. In particular, what are the experiences, motivations, and caregiving impact of two groups of young caregivers of older relatives: children (or caregiving youth) under age 18 and young adult caregivers?
4. What are challenges and open questions that arise from the selected articles and that could suggest future research and policy directions?

The findings related to the last three research questions are framed in a specific conceptual framework, in particular the caregiving stress appraisal model (renamed CSA model) proposed by Yates et al. [55]. This model draws upon both the stress model presented by Pearlin et al. [56] and the appraisal model presented by Lawton et al. [57, 58]. Given that the CSA model [55] is focused on caregivers of all ages, not specifically on young caregivers, it allows us to compare the caregiving experience lived by young (adult) caregivers described in the articles selected by this systematic literature review to the caregiving experienced by caregivers of other ages.

### The conceptual framework

The CSA model [55] explores the relationships between caregiving stressors and caregiver well-being, measured in terms of risk of depression, in a representative community sample of disabled elders and their adult informal caregivers. This conceptual framework, as previously written, is based on the strengths of two different models: the stress model presented by Pearlin et al. [56] and the appraisal model elaborated by Lawton et al. [57, 58]. The model proposed by Pearlin [56] treats stress as stemming from the way caregivers’ lives become organized and the effects of this organization on their self-judgments. According to this approach, stress is a consequence of a process including the socioeconomic characteristics and resources of caregivers and the primary and secondary stressors to which they are exposed.

In particular, primary stressors are hardships and problems anchored directly in caregiving, while secondary stressors are related to two categories: the strains experienced in roles and activities outside of caregiving, and intrapsychic strains, involving the diminishment of self-concepts. Coping behaviors and social support can potentially intervene as mediating factors at multiple points along the stress process. Lawton et al. [57, 58] proposed a conceptual model that adds to the Pearlin findings the importance of the individual appraisal and reappraisal process. According to this, the appraisal of a caregiving stressor is a subjective process accounting for the social, cultural, and economic characteristics of the caregiver.

Furthermore the caregiving is a dynamic process that involves caregivers, care recipients and other psychological and relational aspects. Starting from the strengths of these two models [56–58], the CSA conceptual framework [55] links caregiving stressor, caregiving appraisal and potential mediators to caregiver well-being. In particular, this model is composed of five interrelated factors: 1) the variables of care recipient needs for care or “primary stressor” (i.e. cognitive impairments, functional disability and problems behaviours); 2) caregiver’s primary appraisal (i.e. hours of informal care, that is the response to the care recipient’s health conditions). This process includes both subjective elements (e.g. appraisal of the care recipient’s needs of care) as well as objective ones (e. g. measure of caregiving work); 3) mediators that could change the effects of the stressor on the caregiver’s well-being. These are classified as external (e.g. use of formal services) and internal (e.g. levels of global mastery, quality of the relationship between the caregiver and care recipient, and emotional support available to the caregiver). According to Lawton et al. [57], “mastery” could be defined as a positive view of one’s abilities and the related behavior during the caregiving process; 4) the caregiver’s secondary appraisal (i.e. the caregiver’s perception of being “overloaded”, that is the caregivers’ capability of determining their own feelings about caring); 5) outcomes, i.e. psychological caregiver’s well-being, measured by risk of depression.

According to this model, caregiving is a complex process in which two separate caregiver’s appraisals affect the relationship between the stressors and the outcomes. Hence, the outcomes of the caregiving experience are a subjective process, strictly related to the psychological, social, cultural, and economic characteristics of the subject. Furthermore, CSA model [55] highlights the association between the caregiver’s overload and consequent depression, and the poor quality of the relationship with the older care recipient, especially in case of cognitive and behavioural problems.

According to this conceptual framework, that is based on the experiences of caregivers of all ages, the authors discuss the findings of this systematic literature review in order to highlight differences related to the caregiving experience of young (adult) caregivers.

## Methods

In order to answer the above mentioned research questions, the authors first carried out a methodological appraisal using the Mixed Methods Appraisal Tool (MMAT) [59], and then the contents of each selected article were analyzed thematically. As regards the methodological appraisal, MMAT is designed for the appraisal stage of systematic mixed studies reviews, i.e., reviews that include qualitative, quantitative and mixed methods studies, with the exclusion of non-empirical papers, such as review and theoretical papers. The MMAT includes criteria for appraising the methodological quality of five categories of studies: (a) qualitative studies, (b) randomized controlled trials, (c) non-randomized studies, (d) quantitative descriptive studies, and (e) mixed methods studies. For each study category, MMAT provides two groups of questions: 1) two screening questions aimed at exclude that the paper is not an empirical study and thus cannot be appraised using the MMAT; 2) five questions targeted to evaluate the methodological distinctive specific characteristics of the appraised study.

Each criterion is rated on a categorical scale: yes, no, and can’t tell. A quantitative appraisal score was calculated by applying the scoring system proposed by Pluye and colleagues [60]. According to them, the presence/absence of criteria (yes/no) may be scored 1 and 0, respectively. Thereafter, a ‘quality score’ can be calculated as a percentage: [(number of ‘yes’ responses divided by the number of ‘appropriate criteria’) × 100] [60].

In this systematic review, first and second authors independently appraised the methodological quality of each study; the results of each appraisal were compared and any disagreements were solved through intervention of the third author and discussion among the authors.

Finally, after calculating the above mentioned appraisal score for each article, we synthesize methodological quality results in three different categories:

- Low score= < 35%
- Medium score= from 36 to 70%
- High score= from 71 to 100%

As regards the content analysis, in order to examine characteristics, conditions and needs of young caregivers of older relatives, the content of each article was analyzed adopting the constant comparison technique [61]. According to the latter, each study was read by each researcher independently and the contents are codified in order to highlight concepts that were raised from the study. Then, these codes were constantly compared with the findings of the other selected studies to the purpose of identifying common themes and conceptual categories. At the end of this analytical stage, the researchers compared the outcomes of their independent research in order to identify commonalities and to discuss any disagreement. The categories emerged from the studies were grouped according to their similarities into overarching themes, as shown in the section Results.

### Search strategies

PubMed, Web of Science, Scopus, ProQuest - Psychology Database, CINAHL Complete - EBSCOHost were accessed by the first author (B. D’Amen) on January 27, 2019, for the sake of conducting a comprehensive search using a combination of Boolean operators and terms related with the topic. Given that the focus of this review is the relationship between young and young adult caregivers and older care recipients, two different groups of terms were selected for the identification of the articles. A pilot search was conducted in the selected databases, after which some minor changes were made to correct search words. In particular, for the caregiver category we used the terms: *young caregivers*, *young carers*, *young adult caregivers*, *young adult carers*. Considering the care recipient category, we used the following terms: *older family members*, *older people*, *older adults* and *elderly*.

These two groups of terms were combined in search strings constructed using the Boolean operator “AND”. Following this search strategy, a total of 3,947 articles were identified through database searching, 26 additional articles were added through bibliographic research and researchers’ knowledge. From the 3,973 articles, 1,481 duplicates were identified manually and then removed. Four different eligibility criteria were applied for the selection of the articles. The first one was the age range of young and young adult caregivers. As Joseph et al. [38] stated, there is no single definition of both young and young adult caregiver. In particular, as already mentioned in the Introduction, the analysis of the literature on young and young adult caregivers reveals that there is quite a bit of variation in the definition of the age range of these categories of individuals. For example, Aldridge et al. [28] defined young caregivers as children, adolescents and teenagers under 18 years, while Beach [29] defined them as young people aged 14 to 18 years. Later, Fruhauf et al. [26] defined people aged 7 to 29 as young caregivers. The same happened for the definition of young adults. Dellmann-Jenkins et al. [25] have defined subjects aged 18 to 40 years as young adult caregivers, whilst Becker et al. [19] included in this category those aged 16 to 24.

**Fig. 1:**
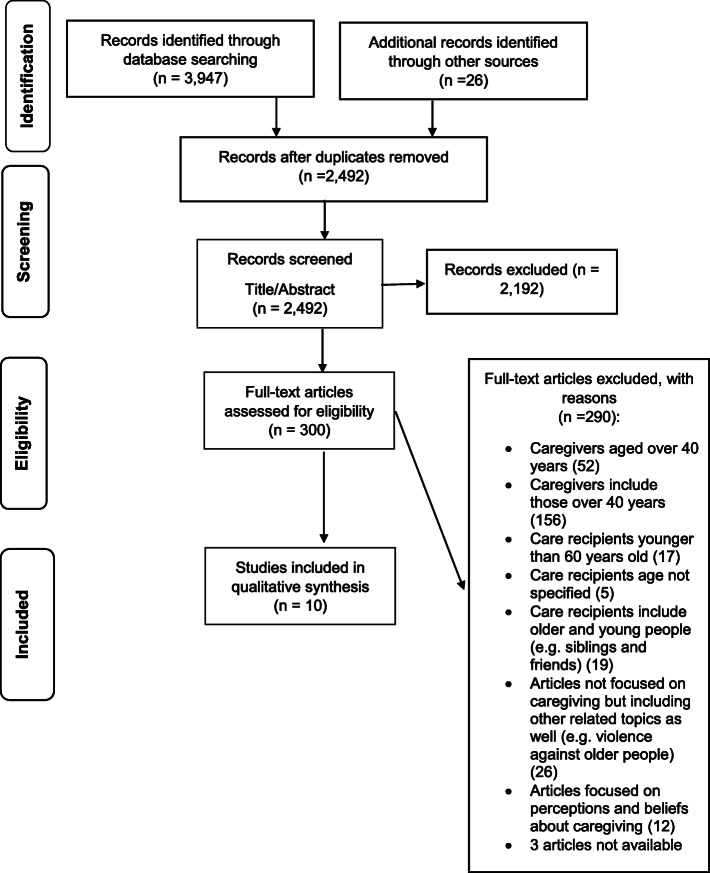
The PRISMA flow chart for reporting the study screening process

Given these differences concerning the age brackets, the authors selected a wide age range, up to 40 years, in order to be as much inclusive as possible. Another eligibility criterion regarded the age of care recipients, set at 60 years onwards. Although there are commonly used definitions of old age (i.e. 65 years old), there is no general agreement on the age at which a person becomes old. Given this lack of a standard numerical criterion, in this literature review we adopted the cutoff point of 60 years to refer to the older population, according to the United Nations [62]. Moreover, the selected articles had to be focused on real life cases of caregiving, which means that, accordingly, studies merely focused on perceptions or beliefs about caregiving were excluded. Finally, this systematic literature review included articles written in English. These eligibility criteria were applied for the screening of the 2,492 studies. Thereafter, 2,192 articles were excluded through the analysis of both title and abstract and 300 full articles were assessed for eligibility. At the end of this selection process, the total number of articles included in the analysis was 10 (Fig. 1).

The three authors carried out the selection process independently: the first author reviewed 2,101 articles, the second author 250 and the third author 141 articles. The results of this selection process were checked by the authors independently. In particular, the screening process carried out by an author, say the first, was checked by one of the other two in order to verify the accuracy of the selection process described in the PRISMA chart [63]. Applying the eligibility criteria, each author ended up with the same results obtained by the colleagues.

## Results

This review includes 10 studies and their main methodological and content characteristics are described in the following section.

### Study characteristics

Concerning the selected studies, six are qualitative, one quantitative and three follow mixed methods. Moreover, three studies adopt a comparative approach. In particular, Dellmann-Jenkins et al. [25] compared adult children and grandchildren as family caregivers; Dellmann-Jenkins et al. [40] explored differences between young adults who were primary caregivers to impaired older relatives and young adults who had yet to take on caregiver roles; Fruhauf et al. [26] analysed caregivers according to age.

Table 1 provides an overview of the main characteristics of the studies: data were extracted from each study by the three authors and the results were collected and classified by the first author.

**Table 1:** Description of 10 Studies of Young and young adult caregivers of older people

Authors (year) [Ref number]	Methodology	Data collection technique	Sample size (n)	Caregiver socio-demographics^**a**^	Type of caregiving role	Care recipient characteristics^**b**^	Findings classified by main themes
Fruhauf & Orel (2008) [26]	Qualitative	Face-to-face semi-structured interviews	34	*Age*: 7 to 29 years *Sex:* 19 females, 15 males *Education:* 5 primary school; 3 middle school; 9 high school; 1 associate degree; 7 had or were finishing bachelor degrees; 7 post-graduate degree *Nationality-ethnicity:* 26 white, 6 black and 2 Latinos *Relationship to care recipient:* grandchildren	1 primary 9 secondary 24 auxiliary	*Age:* 67 to 93 years *Sex:* 27 females, 23 males *Pathology:* cognitive and/or physical limitations	a) Types of activities performed by caregivers b) Feelings related to providing care c) Coping strategies in response to caregiving demands
Fruhauf, Jarrott, & Allen (2006) [5]	Qualitative	Face-to-face interviews	17	*Age:* 21 to 29 years *Sex:* 9 females, 8 males *Education:* all reported having some type of higher education, 11 participants enrolled in college *Nationality-ethnicity*: 14 White, 3 Black *Relationship to care recipient:* grandchildren	1 primary 9 secondary 7 tertiary	*Age:* 72 to 91 years *Sex*: not specified *Pathology*: not specified	a) How young caregivers define their role b) How they make sense of their role c) What they like about their role d) What they do not like about caregiving e) Coping strategies
Dellmann-Jenkins, Blankemeyer, & Pinkard (2000) [25]	Mixed methods	Structured and open-ended interviews	43	*Age:* 18 to 40 years *Sex:* 38 females, 5 males *Education:* 20 high school graduates, 10 attended some college, 13 college graduates *Nationality-ethnicity:* 35 White, 6 Black, 1 Asian American, 1 Native American *Relationship to care recipient*: 20 daughters, 19 granddaughter, 2 sons, 2 grandsons	Primary	*Age:* 60 to 96 years *Sex*: not specified *Pathology*: not specified	a) Frequency and type of assistance provided b) Strains resulting from the caregiving role c) Positive outcomes resulting from the caregiver role d) Caregiver’s use of support sources e) Words of wisdom and recommendation
Orel & Dupuy (2002) [42]	Qualitative	Semi-structured interviews	6	*Age:* 7 to 17 years *Sex:* 4 females, 2 males *Education:* not specified *Nationality-ethnicity:* White *Relationship to care recipient:* grandchildren	Auxiliary	*Age:* mean age 78.6 years *Sex:* not specified *Pathology:* limitations in physical and/or cognitive functioning	a) Coping strategies b) Positive outcomes of caring c) Negative outcomes of caring d) Recommendations
Beach (1997) [29]	Qualitative	Semi-structured interviews	20	*Age:* 14 to 18 years *Sex*: 11 females, 9 males *Education:* the majority completed high school education *Nationality-ethnicity: 13* White, 3% Black, 2% Native Americans, 2% Pacific islanders *Relationship to care recipient*: 4 children, 12 grandchildren and 4 nieces/nephews	Auxiliary	*Age:* mean age 69 years *Sex:* not specified. *Pathology:* Alzheimer’s disease or dementia	a) Positive impact on family relationships, specifically with siblings b) Greater empathy for older adults c) Greater intimacy within mother/adolescent relationship d) Selection of friends among peers
Dellmann-Jenkins & Brittain (2003) [40]	Mixed methods	Multiple-choice questionnaire; Six open-ended questions	80	*Age*: 18 to 40 years *Sex:* 68 females, 12 males *Education:* 7 primary school, 35 high school, 28 undergraduate degree, 4 trade vocational degree, 6 master degree *Nationality-ethnicity:* 70 White*,* 2 Native Americans, 6 Black *Relationship to care recipient*: not specified	Primary	*Age:* 64 to 91 years *Sex*: not specified *Pathology:* physical and/or cognitive impairments	a) Reasons for caring b) Type of support provided to care recipient c) Negative and positive outcomes of caring d) Need for support
Dellmann-Jenkins, Blankemeyer, & Pinkard (2001) [64]	Mixed methods	Multiple choice questionnaire; Open-ended questions; Close-ended questions	50	*Age*: 18 to 40 years *Sex: 45* females, 5 males *Education*: 1 completed primary school, 24 high school graduates, 3 attended college, 9 enrolled in college, 11 college graduates, 3 graduate degree *Nationality-ethnicity:* *43* White, 3 Black, 2 Asian Americans, 2 Native Americans *Relationship to care recipient:* 21 daughters, 3 sons, 16 granddaughters, 2 grandsons, 4 daughters-in-law, 1 granddaughters-in-law, 1 great-granddaughters, 2 nieces	Primary	*Age:* 60 to 96 years *Sex:* not specified *Pathology:* 81% have more than one cognitive or physical limitation	a) Differentiation from the family of origin b) Establishing intimate relationships c) Career development
Hamill (2012) [41]	Quantitative	Structured telephone inteviews	29	*Age:* 11 to 21 years *Sex:* 21 females, 8 males *Education*: not specified *Nationality-ethnicity:* 7 families were Mexican Americans (8), and the remaining were whites (21) *Relationship to care recipient:* grandchildren	Auxiliary	*Age:* Mean age 79.29 years (SD=8.28) *Sex:* not specified *Pathology:* Alzheimer’s disease	a) Grandchildren as auxiliary caregivers b) How is caregiving related to adolescent development c) How is caregiving related to attitudes to long-term care
Orel, Dupuy, & Wright (2004) [27]	Qualitative	Semi-structured interviews	6	*Age:* 7 to 17 years *Sex:* 4 females, 2 males *Education:* 2 primary school, 4 secondary school *Nationality-ethnicity:* White *Relationship to care recipient:* grandchildren	Auxiliary	*Age*: 71 to 91 years *Sex:* 3 females, 3 males *Pathology:* 2 hypertension arthritis, 1 vascular dementia arthritis, 1 hypertension diabetes arthritis, 1 Alzheimer arthritis, 1 Amyotrophic lateral sclerosis (ALS)	a) Grandchildren’s perception of disease b) Grandchildren’s perceptions of care recipient c) Caregiving activities performed by grandchildren d) Non-caregiving activities with grandparents e) Feelings related to providing care f) Grandchildren’s perceptions of parents’ caregiving responsibilities
Blanton (2013) [65]	Qualitative	In-depth interview	10	*Age:* 17 to 30 years *Sex:* not specified *Education:* not specified *Nationality-ethnicity:* 8 White, 1 Black, 1 Hispanic *Relationship to care recipient:* grandchildren	Auxiliary	*Age:* not specified *Sex*: 5 females, 5 males *Pathology:* 8 were both physically frail and cognitively impaired, 2 only physically frail	a) The diversity and continuity of the nature of intergenerational relationships in families b) Nature of involvement in the family caregiving process c) Feeling of being caught in the middle or pulled in various ways

^a^Age, sex, education, nationality-ethnicity, relationship to care recipient

^b^Age, sex, pathology

The selected studies cover a time ranging from the late nineties to 2013. The sample size ranges from a minimum of six to a maximum of 80 subjects; three articles included in the sample even young caregivers’ parents [27, 41, 42], whilst the others were focused only on young caregivers. Concerning the characteristics of caregivers, the articles provide evidences on 255 young individuals, 190 females and 65 males. The age ranges from 7 to 40 years. The selected articles considered different types of caregiving, categorized on the basis of the amount of assistance provided and the related burden. Three studies [25, 40, 64] were focused on primary caregivers [4, 6], four [27, 41, 42, 65] on auxiliary ones [9], and three articles [5, 26, 29] included different types of caregivers, with a prevalence of auxiliary ones. Out of a total of 255 caregivers included in the selected studies, 53% were primary caregivers, 15% secondary [8], 3% tertiary and 29% auxiliary [26].

On the other side, the number of care recipients was not always specified, so it is not possible to quantify the cases of multi-caregiving. The age of the cared-for older people ranges from 60 to 96 years and the sex was specified only in three articles [27, 65], whilst in the other studies this was not reported, or can be partly deduced from the relationship between caregiver and care recipient. Concerning the type of pathology/health issues, two articles [5, 25] did not provide information, whilst two more [29, 41] were focused on care recipients with Alzheimer’s disease or dementia. The remaining studies concerned cared-for older people having various pathologies, cognitive or physical limitations.

### Methodological and quality appraisal

As previously written, the methodological appraisal was carried out through the MMAT, the critical appraisal tool developed by Hong and colleagues [59].

According to the appraisal process described in the section methods, the results related to the methodological quality of the selected articles are summarized in the table below (Table 2).

**Table 2:** Results of the methodological appraisal

First Author (Year) [Ref number]	Type of study	Screening	Qualitative	Quantitative	Mixed methods	Total	Number of appropriate criteria	Quantity score^**a**^	Score category
Dellmann-Jenkins (2001) [64]	Mixed methods	2	5	4	5	16	17	94%	High
Beach (1997) [29]	Qualitative	2	5	0	0	7	7	100%	High
Hamill (2012) [41]	Quantitative	2	0	3	0	5	7	71%	High
Orel (2004) [27]	Qualitative	2	5	0	0	7	7	100%	High
Blanton (2013) [65]	Qualitative	2	2	0	0	4	7	57%	Medium
Fruhauf (2008) [26]	Qualitative	2	5	0	0	7	7	100%	High
Fruhauf (2006) [5]	Qualitative	2	5	0	0	7	7	100%	High
Orel (2002) [42]	Qualitative	2	5	0	0	7	7	100%	High
Dellmann-Jenkins (2003) [40]	Mixed methods	2	5	4	5	16	17	94%	High
Dellmann-Jenkins (2000) [25]	Mixed methods	2	5	4	5	16	17	94%	High

^a^ Quantitative score: [(Total/Number appropriate criteria) ×100] [60]

Considering the score system, almost all selected articles had a high methodological quality, except for a qualitative study [65] that got a medium score. The main critical aspect of quantitative and mixed method studies concerned the sample size, considered too small to be representative of the target population. The limited number of individuals involved in these studies was justified by the authors of the articles with the difficulty of reaching young caregivers, due to the lack of awareness and knowledge around this issue. In their opinion, therefore, the small sample size should be considered as a limitation concerning the target population rather than a weakness of the research studies design in itself.

As regards the qualitative studies, the structure of the interviews was not always explained, and in the medium score article the description of the coding and analytical process was not so accurate, so it was not possible to understand the bias that could come from researcher beliefs.

### Content and findings analysis

The review highlighted four major themes: aspects of the caregiving relationship, effects of caring, coping strategies and recommendations for services, policy and research. Each theme has been organized in sub-themes that are dealth with in depth hereunder.

### Aspects of the caregiving relationship

#### Motivations

The motivations pushing a young person to take on the care of an older relative are multiple and often interconnected, e.g. affection and bond with the older person [5, 25] and the will to avoid his/her institutionalization (e.g. in nursing homes) [25, 40]. In some cases, contextual/familiar conditions, including the lack of availability of assistance from more adult relatives [25, 40] or being childless [25, 40], can be drivers of youth caregiving. Considering the CSA model [55] adopted as conceptual framework of this review, these findings confirm the relevance of the personal motivations and of the subjective appraisal in the caregiving relationship also for the youngest. However, given that these motivations could be often multiple, in the case of young caregivers it is important to adopt a wider definition of the first and the second factors, that in the above mentioned model are related to the “variables of care recipients’ needs (or primary stressor)” and to the “primary appraisal”, by considering the role of external factors, such as the contextual/familiar conditions, that in some cases might have a role in the caregiving relationship and its effects for the youngest. Usually, the quality of the relationship with parents is not an aspect influencing the willingness to provide care, but young people often help simply because there is a practical need for assistance [41]. However, in the case of primary caregivers, there are differences related to their roles: grandchildren seem to be driven more by feelings of attachment, while children have feelings of filial obligation towards the cared-for person [25].

#### Perceptions and meaning

Caregiving is perceived as an experience for returning the care that older people had provided in the past to the youngsters [5]. In the case of auxiliary caregivers, those most involved in caring tasks were individuals with higher levels of cohesion and lower levels of conflict in intergenerational relationships, coupled with a positive history of interactions with their grandparents [65]. These findings suggest us to deepen the CSA model [55], by considering the quality of the relationship not only as “mediators” of the caregiving relationship, that might mitigate the impact of the stress, but also as a “driver” that contributes to foster the caregiving relationship between young or young adult caregivers and older patients.

Caregiving has also been defined as a case of role reversal, in which young caregivers find themselves caring instead of receiving care [5]. As for the perception of the subjects involved in the caregiving relationship, one study [27] highlighted how grandchildren recognize themselves as auxiliary caregivers, while mothers are perceived as engaged in more caregiving activities than fathers. The care recipients are accepted and understood by young caregivers because of their illness, though these perceptions are mediated by the information given by parents. Accordingly with the CSA model [55], in particular with the process of appraisal, these findings confirm the relevance of the subjective perceptions of the caregiving role experienced by the youngest as factors involved in determining the sense of the overall caregiving relationship. Thus, the latter is not a simple response to specific care needs, but includes subjective meanings that could play a significant role in providing care and in determining its effects.

#### Experience of caregiving and activities performed

The analysis of selected articles showed that young caregivers carry out a wide range of activities, ranging from helping the cared-for older people in performing ADLs [41, 65] and instrumental activities of daily living (IADLs) [41], to companionship, assistance for shopping, personal hygiene, meal preparation [40] and emotional support [65]. Sometimes caregiving activities are based on parental directives [26, 27], although in some other cases the youngest take the initiative to carry out certain activities that can be particularly appreciated by the care recipient, such as shaving of the legs [26, 27]. Even though the variety of caregiving tasks is dependent on both grandchildren’s developmental/emotional condition and care recipients’ caring needs [27], young people provide more care when they are more attached to grandparents and when their parents experience a greater care burden [41].

### Effects of caregiving

#### Negative effects

The effects of caregiving are analyzed from the perspective of the current conditions experienced by young caregivers and in some cases, too, from the perspective of the implications for future life [5, 40–42]. With regard to negative aspects stemming from the caregiving relationship, compared to the contingent living conditions, young people report feelings of anxiety, depression [26], anger and/or resentment [27], sadness mingled with compassion [27], and a sense of guilt, deriving from the fact that one often wanted to do something else [26].

Many young caregivers declare that they feel fear, a feeling often resulting from health conditions of the care recipient [5, 25, 27, 64] and the feeling that these may worsen. These feelings can be accompanied by frustration, generated in young people by tasks that go beyond their skills [26]. Youngsters usually state that, although they know the technical terms related to the disease of the care recipient, they find it difficult to understand them [27]. Moreover, they do not understand what they must do in a dangerous situation [26] and they complained of a lack of information on the care recipient’s health condition [27]. Furthermore, young caregivers do not know the level of knowledge and skills required to provide care properly [41]. Accordingly with the CSA model [55], these findings confirm the relevance of the “primary appraisal”, focused on the care recipient’s health conditions, and the sense of “mastery”, considered as internal resources and “mediator” of the caregiving effects, in determining negative outcomes of the caregiving relationship. Frustration can be often caused by a decrease in free time to dedicate both to themselves [40] and to other relationships [5, 25, 42], including those with their own peers [42] and with other family members [25], which could make it difficult to establish intimate relationships [25, 64]. Concerning young adult working caregivers, even the effects of caregiving in the professional sphere are manifested in feelings of frustration, whose origin is the lower possibility of career advancement, mobility [5, 25, 40, 64], and greater absenteeism from work [25, 40].

The negative effects of caregiving also affect self-image, with the perception of being different from other friends and relatives [5], and the feeling of experiencing a premature role reversal [5, 64]. In addition, caregiving involves a greater difficulty in differentiating from the family of origin [64], an important issue for the construction of individual autonomy whose negative impact on the subsequent marital quality and career choices has been documented [66, 67]. Caregiving has also particularly negative effects on the future life of young caregivers, leading them to develop negative views and feelings about ageing [42]. Particularly important is the role of fathers, in the construction of a sense of social responsibility: when the father provides care, young caregivers might develop less social responsibility and a more negative attitude towards assistance [41].

#### Positive effects

As for the positive aspects associated with care, young caregivers experience an improvement in their self-image, with a greater awareness of their abilities [5, 25, 26, 40, 64, 65], feelings of gratification and satisfaction [42, 65], and the acquisition of new skills [26, 42]. Particularly positive effects on the relational context in which young caregivers experience benefits have been observed not only in the relationship with older people but also with other individuals [5, 25, 29, 40, 42, 64]. Moreover, one study [29] underlines that providing assistance can have a positive impact on family relationships, especially with siblings, and greater intimacy within the mother/adolescent relationship [40].

The caregiving relationship can be at the origin of new relational possibilities: some young caregivers, in fact, to cope with the lack of time and not to deprive themselves of meaningful relationships, include friends in their daily care tasks/routines [5, 26]. In these cases, caregiving is transformed from a factor of social isolation to an opportunity for integration by sharing personal life challenges with peers. Particularly positive effects concern the improvement of some behavioural characteristics, since caregiving can make young people wiser and patient [5]. Providing assistance can positively predispose them to provide care in the future as well, to their partner or children [5, 41], and give them a more positive representation of long-term care for older parents [41] and greater sensitivity to ageing issues [5]. These findings suggest that positive effects of caregiving could be related to an active role of the youngest in managing the outcomes of the caregiving process, so the sense of “mastery” stated by the CSA model [55] is confirmed as an important mediator in reducing distress and in fostering the caregivers’ well-being.

### Coping strategies

To mitigate the negative effects of caregiving, young caregivers could develop different strategies: the use of positive memories with care recipients [26, 42], the minimization of their health conditions [26], positive self-evaluation of one’s role as caregiver, and humour [42]. The stress from the caregiving relationship is also managed through the adoption of particular habits, such as, for example, sports and religious activities [5, 26, 42], and by receiving support from friends [5].

#### Use of services and needs for support

Most young caregivers receive informal support from family, friends, neighbors and the church. Formal support is less frequent and concerns health services and participation in mutual help groups. Particular barriers for the use of formal services are represented by the lack of both economic resources and information regarding available services [25].

Concerning the needs expressed by young caregivers, the possibility of receiving emotional support from other caregivers of the same age, low-cost health services for older people, and their transport to facilitate daily activities [25, 40], are particularly relevant. In particular, according to the CSA model [55], these findings confirm the relevance of emotional support as a mediator factor able to reduce the negative outcomes of the caregiving relationship. Only one among the selected papers [26] compared young grandchildren (aged 7–17) and adult children (aged 21–29) by focusing on caregiving outcomes and caregivers’ needs. This study suggests that younger grandchildren experienced more frustration, anger, guilt, anxiety while developing more behavioral (i.e. avoidance, outside activities) and cognitive coping strategies (i.e. focusing on positive memories, denial, humor) than adult grandchildren. Moreover, the different developmental stage that young and adult grandchildren were living influenced their need, e.g. young grandchildren needed help for understanding what the care recipient’s health condition may entail and adult grandchildren needed more intimacy, as they were in a phase of life in which it is important to build up intimate romantic relationships.

#### Recommendations for services, policy and research

The analysis of the selected articles highlighted implications for research and support services, in particular specific service delivery recommendations. Concerning formal services, it would be desirable to promote the creation of caregiver support groups for the whole family [26]; this support should be established within the educational system and facilitated by school counsellors [42] or offered through existing ageing social service providers [27]. To facilitate the use of services, it would be useful to carry out a need assessment, in order to propose targeted and effective interventions, and to promote a greater knowledge of any support groups present on the territory, especially if they are free [25].

These actions should help the management of different types of stressful factors, including: lack of time to develop relationships, difficulties in managing married life, managing both early career difficulties and psychological discomforts arising from premature role reversal [25]. Caregiver support groups and training workshops specifically designed for multigenerational caregiving families are needed. The latter, for example, would assist parents in explaining grandparents’ physical/cognitive decline to their children [27]. Although young caregivers need to be recognized, identified and supported as a distinct group of vulnerable children [26], information on the impact of caregiving should also be disseminated in the clergy, which appears to be a particularly significant source of spiritual support [25]. Furthermore, workplaces should also have specific counsellor services and flexible work opportunities to support young caregivers in building their professional lives and to better reconcile work and care responsibilities [25].

## Discussion

The studies included in this review analysed the experience of young and young adult family caregivers of older relatives, who represent an under-investigated category of family caregivers [21].

The mainly qualitative approach and research designs of the reviewed studies focused on small collectives give evidence of the scholars’ need for drawing the young family caregivers’ profile and the caregiving dynamics they experience. Given the different role of caregivers included in the selected studies (i.e. primary, secondary, auxiliary and tertiary), this literature review allows us to capture the experience of young and young adults caregivers of older people from different perspectives. Moreover, the reviewed studies have explored the practical aspects of the caregiving relationship (tasks performed, motivations, meaning, coping strategies), and the amount of care provided, thereby confirming the classification of young and young adult caregivers into primary [19, 22, 23] and secondary/auxiliary [20, 21].

The comparative approach adopted by three studies [25, 26, 40] allowed us to better understand the experiences and the different effects of caregiving connected to the role played by young and young adult caregivers in the family environment (i.e. adult children vs grandchildren or caregivers vs non-caregivers), and to their age [26].

In accordance with the literature [16, 17], the selected studies showed that among young family caregivers, females are more involved than males in caregiving activities.

As far as the impact of caregiving is concerned, this review increases the knowledge about the impact of caregiving on young caregivers’ psychological health [45, 47] and social life [28, 49, 50]. Accordingly with the literature [68], this review showed that caregiving activities can have, even simultaneously, a positive and a negative impact on the youngsters’ different life realms. For example, some studies highlighted an improvement in self-image [5, 25, 26, 40, 64, 65], and one of these [5] even reported a worsening on it. Some studies underlined negative effects on social life in and outside the family environment [5; 25, 64, 42], while others recorded positive ones [5, 26].

Moreover, this review allowed us to raise the specificities of caregiving relationship between young people and older relatives in comparison to the caregiving experience of adult caregivers, as conceptualized in the CSA theoretical framework [55]. In this regard, the results suggest that the CSA model [55] is just partly applicable to young caregivers for reasons that are mainly due to the young age of the caregivers. In particular, according to this model indeed, the subjective appraisal of the elder’s need for care determines the amount of care which the caregiver thinks it is to provide, so assuming that caregivers are able to appraise the care recipient’s disability and the more suitable response to his/her needs. Conversely, young caregivers may not be able to make a realistic appraisal of the care needs, due to their young age and the dearth of experience and knowledge. Moreover, the CSA model [55] considers the “overload” a secondary appraisal, so assuming that caregivers are able to identify their level of overload by assessing their own situation and their feelings about caring. This perspective seems to be not appropriate for describing the experience of young caregivers and their level of self-awareness. Furthermore, given that this review highlights that the young caregivers provide more care when their parents experience greater care burden [41], the role of the parent’s burden in providing care should be taken into account for determining the hours of informal care (“primary appraisal” in the CSA model [55]) and the young caregivers overload (“secondary appraisal” in the CSA model [55]). Conversely, a common point of CSA model [55], which considers adult family caregivers, and the results of this review, focused on young family caregivers, lies in the quality of the relationship between the adult family carer as an element which can affect the perception of the caregiving experience and so the caregivers’ well-being.

The youngsters, even knowing the terms related to the cared-for illness and responding to the parents’ requests for help, often do not fully understand pathology risks [26]. This exposes them to greater feelings of inadequacy, fear, and frustration [26] than adult caregivers on whom they often rely for information acquisition [27], thereby confirming the findings of Järkestig-Berggren et al. [46]. These findings suggest to improve the Yates model [55] by highlighting the relevance of this mismatch between the care demand and the young caregivers’ knowledge and emotional resources as factors that could play an important role in determining the caregiving outcomes. Moreover, given that the awareness of this mismatch could encourage adult family members to reshape the requests to young caregivers, this could be an important factor able to reduce the young caregivers burden. Despite these theoretical consequences, this mismatch should be taken into account by professionals responsible for health and social care services and policy makers, in order to provide training interventions and support policies for young and adult caregivers. A relevant aspect that has to be added in the CSA model [55] is the relevance of supporting young caregivers in understanding the care recipient’s illness. This is related to a communication aspect that could be included in the set of factors stated by the CSA model [55] and directed at how caregivers appraise the needs for care (primary appraisal). Furthermore, as stated by the CSA model [55] and confirmed by Chappell et al. [69] the perceived social support, such as emotional support from family and friends, is strongly related to caregiver’s well-being and unrelated to the burden. Given that the emotional support is relevant also for young caregivers [25, 40], providing interventions that address this aspect or specifically focused to develop skills to elicit desired emotional support from family and friends is an important aspect for improving caregivers’ quality of life even with caregiving burden in their lives.

Finally, in light of the insights on the role of the father caregiver in influencing children’s perception of caregiving experience and social responsibility [41], the relationship of the caregiver with other family members might provide an interesting reading key to identify those “family-embedded” factors that contribute to determine the effects of caregiving on young people in the present as well as in the future (e.g. the willingness to keep on providing care).

### Weaknesses of the reviewed studies and suggestion for future research

A key-point in research on young caregivers is the lack of a homogeneous definition of the age range for identifying a young caregiver and of a categorization of different sub-groups of caregivers according to their age. The latter might indeed help scholars capture how motivation to care, needs for help and coping strategies change in different phases of life.

Conscious of this general bias, the selected studies were not without specific weaknesses. The first limitation is the small sample size and the co-presence of individuals carrying out different caregiving activities. Thus, future studies should consider whether to include in samples youngsters playing the role of primary, or secondary or auxiliary caregivers, or whether to include all these categories, and how to control the confounding factors that each role entails (e.g. amount of care and caregiving activities).

Secondly, in the selected studies the illness of the care recipient was not sufficiently analysed as a factor influencing caregiving activities and relationships. Hence, particular attention in the design of future studies should be directed to the impact of different types of care recipient’s illness on the youngsters’ perception of the assistance provided. Given that research has shown specific difficulties to care for older people with dementia [70], it could be interesting to understand whether cognitive and physical impairment generate different feelings and coping strategies among young caregivers [26].

Another aspect that could be further investigated concerns the context of care i.e. the grandchildren-grandparents housing condition. Cohabiting with the care recipient, indeed, was not within the sample inclusion criteria of the majority of the reviewed studies, and only one article treats this topic [27]. Nevertheless, in light of the articles cross-reading, living in a multigenerational environment where there is a grandparent in need of care seems to be a driver for involving the youngest in the caregiving activities, especially if the grandparent(s) suffer from cognitive impairment, dementia, Alzheimer disease [41]. Thus, further studies that deepen the association between the context of care and the involvement of the young family members in the caregiving activities are welcome.

Moreover, the sex gap is worthy of further study to understand possible differences in reacting to adverse or difficult situations due to care between girls and boys. The supports available for and needed by young caregivers were still inadequately explored. Conversely, the analysis of available public support services allows us to understand to what extent welfare state measures are able to identify and help young family caregivers.

The reviewed studies included in the samples mostly white individuals. Recent literature states that belonging to black and minority communities can be a driver for being a young caregiver [47] and underlines the influence of cultural patterns on the construction of the meaning of care by young family caregivers [71]. Thus, it would be important to develop studies involving different minority groups, as well as different countries, since all selected studies have been carried out in the U.S.

One study [25] highlighted how the perception of care burden can change in accordance with the type of relationship and of emotional bond with the care recipient (e.g. children or grandchildren of the cared-for person). Thus, more studies on the influence of the relational bond on the young caregivers’ experience would be welcome. Moreover, research based on larger and longitudinal samples would allow us to analyze how the caregiving relationship evolves over the years and the effects that it might produce on young people even after the death of the care recipient. In this regard, it is interesting to highlight that none of the reviewed studies investigates the association between the duration of care and the effects of caregiving. This is an aspect which deserves more attention from future studies.

It would also be important to plan research studies able to investigate the cases in which the caregiver provides care to more than one person, including the support offered to primary caregivers [65]. Furthermore, in light of the contradictory findings concerning the caregiving outcomes on youngsters’ physical, psychological and social well-being, future studies should search for possible factors that can favor the predominance of negative or positive effects and the extent to which the latter can compensate the former.

### Suggestions for teachers, health and social workers

Particular attention should also be paid to spread awareness and knowledge of this phenomenon among health and social service professionals, in order to facilitate the identification of young caregivers, offer them support, and make their recruitment by researchers easier. These actions should be taken into account for improving the young caregivers’ well-being and should be added to interventions focused on developing skills to elicit desired emotional support from family and friends, as argued by the CSA model proposed by Yates et al. [55] and Chappell [69]. An important topic for future interventions on young caregivers of older relatives will be to explore ways to help their self-identification as caregivers. Furthermore, future studies should consider the welfare and healthcare systems where the caring relationship took place, since this analysis could help us understand to what extent the reasons for caring are driven by exogenous and systemic factors (e.g. availability of services, tailored policies, informal care networks) or by personal ones (e.g. resilience, psychological sources).

### Limitations

The searches of the articles were limited to five databases (PubMed, Web of Science, Scopus, ProQuest - Psychology Database, CINAHL Complete - EBSCOHost) accessed by the first author over a specific period of time (January 2019). In order to be more inclusive, the authors added studies selected through a bibliographic research. Moreover, the terms and the Boolean operator applied might not be comprehensive: in particular, specific terms related to the subjects, such as “senior”, “grandparent”, “grandchildren”, “children”, and terms related to a specific pathology, were not included as search terms. Given that our search could not cover all the terms related to the main topic of this article, the results of this systematic review could be not exhaustive and, unknowingly and unintentionally, some papers might have been omitted. Another factor that contributed to the reduction of the included studies is the age ranges adopted for defining young (adult) caregivers and older care recipients. In particular, some studies did not exclusively concern the relationship between young (adult) caregivers and older care recipients and include caregivers older than 40 years or care recipients younger than 60 years. Finally, a further limitation concerns the absence of studies located in the USA, Canada, Europe and Australia among the selected manuscripts. In fact, although the interest of the social sciences for intergenerational relations was born in the USA at the end of the last century and it spread to Europe since the early 2000s, few studies have focused on caregiving relationships within the dyad young grandchild-grandparent (aged 60 and over) i.e. mainly between grandchildren and grandparents [72–76]. This represents a limit of this systematic literature review however not attributable to the authors who explored valuable and rich databases.

## Conclusions

The phenomenon of young and young adult caregivers of older family members in need of care is still largely uncovered. Further reflections for finding “shared definitions” are needed, as well as quantitatively large sample and mixed-methods studies for deepening the different aspects of caregiving relationships that have been studied so far. In fact, as stated by the CSA model [55], caregiving is itself a complex experience whose effects and meanings go beyond the mere dyadic relationship between young caregiver and old care recipient, including the whole family. Considering the CSA model [55], assumed as theoretical model for framed the findings of this review, this article adds some specific factors related to the young age of the caregivers, such as the role of the parents’ burden in determining the young caregivers’ load and the difficulties presented by young caregivers in understanding the care recipient’s illness. These evidences could improve the CSA model [55] in order to better analyze the young caregivers’ well-being.

## Data Availability

The data are available in the articles included in the review.

## Abbreviations

ADLs: Activities of Daily Living
CSA: Caregiving stress appraisal (model)
IADLs: Instrumental Activities of Daily Living
MMAT: Mixed Methods Appraisal Tool
PRISMA: Preferred Reporting Items for Systematic Reviews and Meta-Analysis
