# Correction to: Intergenerational caring: a systematic literature review on young and young adult caregivers of older people

**DOI:** 10.1186/s12877-022-02804-2

**Published:** 2022-03-22

**Authors:** Barbara D’Amen, Marco Socci, Sara Santini

**Affiliations:** Centre for Socio-Economic Research on Aging, IRCCS INRCA - National Institute of Health and Science on Aging, Via Santa Margherita 5, 60124 Ancona, Italy

**Correction to: BMC Geriatr 21, 105 (2021)**

**https://doi.org/10.1186/s12877-020-01976-z**

After publication of this article [1], the authors reported that the sections “Funding” and “Acknowledgments” have to be corrected, by mentioning the EU-H2020 Me-We project (https://me-we.eu/the-project/), as follows:

- Funding: “The study was conducted in the framework of the ME-WE project, which has received funding from the European Union’s Horizon 2020 research and innovation programme under grant agreement No 754702. This work was also partially supported by Ricerca Corrente funding from Italian Ministry of Health to IRCCS INRCA. This funding body did not play any role in designing the study nor in data collection, analysis and interpretation, nor in writing this paper”.
- Acknowledgements: “The authors would like to thank researchers, professionals and organisations who contributed to the ME-WE project. In particular, the authors thank the partner organisations of the ME-WE project (www.me-we.eu), namely: Linnaeus University (Sweden), coordinator; Eurocarers (Belgium); University of Sussex (United Kingdom); Carers Trust (United Kingdom); Kalaidos University of Applied Sciences (Switzerland); Netherlands Institute for Social Research (Netherlands); Vilans (Netherlands); National Institute of Health and Science on Ageing (IRCCS INRCA) (Italy); Anziani e Non Solo (Italy); University of Ljubljana (Slovenia).”

The original article [1] has been updated.
